# 9-Chloro­methyl-9-[(9*H*-fluoren-9-yl)meth­yl]-9*H*-fluorene

**DOI:** 10.1107/S1600536808012117

**Published:** 2008-05-03

**Authors:** Qun Shen, Shu-Qiang Yu, Bin-Bin Hu, Ping Lu

**Affiliations:** a Department of Chemistry, Zhejiang University, Yuquan Campus, Hangzhou 310027, People’s Republic of China

## Abstract

In the title compound, C_28_H_21_Cl, the dihedral angle between the two fluorene ring systems is 71.97 (4)°. There is an intra­molecular C—H⋯Cl hydrogen bond. In the crystal structure, the centroid-to-centroid distance between stacked fluorene ring systems is *ca* 4.22 Å, which indicates that there are no π–π stacking inter­actions between them.

## Related literature

For general background, see: Chun *et al.* (2003 ▶); Kim *et al.* (1998 ▶); Muller *et al.* (2003 ▶); Saragi *et al.* (2004 ▶).
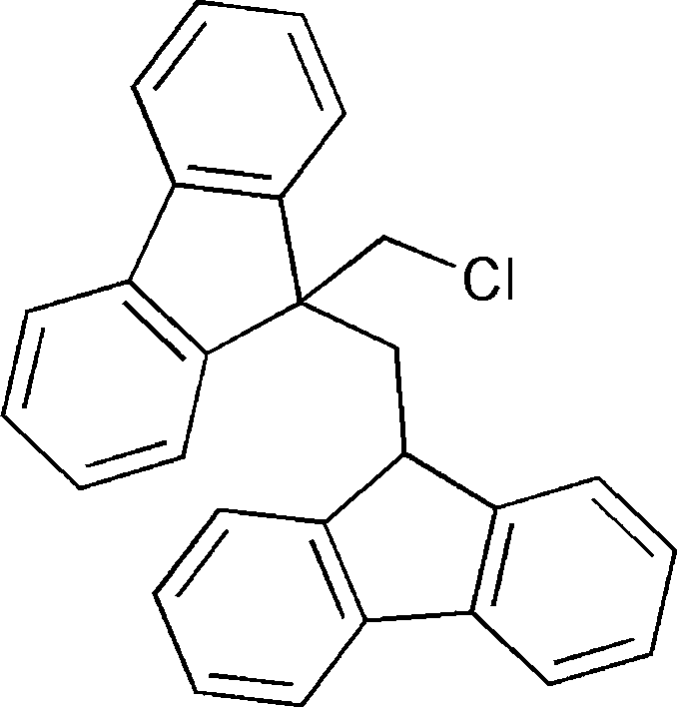


## Experimental

### 

#### Crystal data

C_28_H_21_Cl
*M* *_r_* = 392.90
Monoclinic, 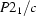

*a* = 8.4346 (17) Å
*b* = 26.368 (5) Å
*c* = 9.1162 (18) Å
β = 94.08 (3)°
*V* = 2022.3 (7) Å^3^
*Z* = 4
Mo *K*α radiation
μ = 0.20 mm^−1^
*T* = 298 (2) K
0.35 × 0.29 × 0.22 mm

#### Data collection

Bruker SMART 1000 CCD area-detector diffractometer
Absorption correction: none
16094 measured reflections
3646 independent reflections
2747 reflections with *I* > 2σ(*I*)
*R*_int_ = 0.025

#### Refinement

*R*[*F* ^2^ > 2σ(*F* ^2^)] = 0.040
*wR*(*F* ^2^) = 0.133
*S* = 1.08
3646 reflections
263 parameters
H-atom parameters constrained
Δρ_max_ = 0.22 e Å^−3^
Δρ_min_ = −0.26 e Å^−3^

### 

Data collection: *SMART* (Bruker, 2001 ▶); cell refinement: *SAINT* (Bruker, 2001 ▶); data reduction: *SAINT*; program(s) used to solve structure: *SHELXTL* (Sheldrick, 2008 ▶); program(s) used to refine structure: *SHELXTL*; molecular graphics: *SHELXTL* software used to prepare material for publication: *SHELXTL* and *publCIF* (Westrip, 2008 ▶).

## Supplementary Material

Crystal structure: contains datablocks global, I. DOI: 10.1107/S1600536808012117/xu2420sup1.cif

Structure factors: contains datablocks I. DOI: 10.1107/S1600536808012117/xu2420Isup2.hkl

Additional supplementary materials:  crystallographic information; 3D view; checkCIF report

## Figures and Tables

**Table 1:** Hydrogen-bond geometry (Å, °)

*D*—H⋯*A*	*D*—H	H⋯*A*	*D*⋯*A*	*D*—H⋯*A*
C27—H27*A*⋯Cl1	0.97	2.68	3.075 (2)	105

**Fig. 1.**
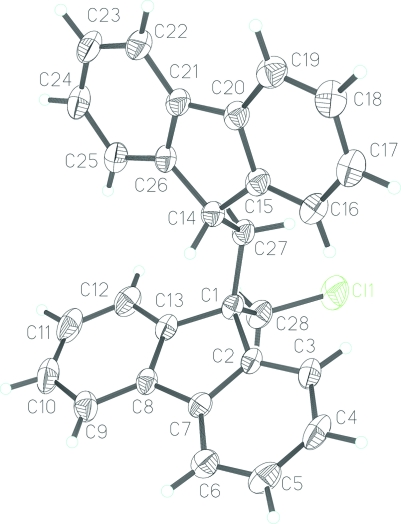
The molecular structure showing the atom-numbering scheme. Displacement ellipsoids are drawn at the 30% probability level. H atoms are shown as small spheres of arbitrary radii.

**Fig. 2.**
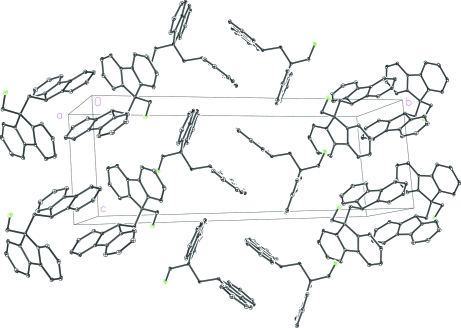
Partial packing view of the title compound. H atoms are omitted for clarity.

## supplementary crystallographic information

### Comment

Fluorene derivatives, have attracted much attention due to their potential utilities in organic lightemitting devices (Muller *et al.*, 2003), organic phototransistors (Saragi *et al.*, 2004), nonlinear optics (Kim *et al.*, 1998) and photochromic materials (Chun *et al.*, 2003). The title compound (hereinafter abbreviated to fmcf) is one of fluorene derivatives.

The asymmetric unit of the title compound contains one fmcf molecule (Fig. 1). The chloromethyl group is attached on the C-9 position of one fluorene ring. Two fluorene rings are linked together through a methylene carbon atom, and the dihedral angle between the two fluorene rings is 71.97 (4)°. There is intramolecular C–H···Cl hydrogen bond with distance of 3.075 (2) Å (Table 1), while the intermolecular C–H···Cl contacts are of 3.573 (2) Å, which is not viewed as C–H···Cl hydrogen bond. The centroid to centroid distance between stacked fluorene rings is *ca*. 4.22 Å, which is very long and prevents π···π stacking (Fig. 2). All bond lengths and angles are normal.

### Experimental

All chemicals were of analytic grade quality obtained from commercial sources and used as received, unless stated otherwise. To a solution of fluorene (1.66 g, 10 mmol) in dry THF (40 ml) was added a hexane solution of n-butylithium (4 ml, 2.5 *M*, 10 mmol) under nitrogen at -78 °C, the mixture was stirred for 1 h. A solution of PCl_3_ (2 mmol) in THF (10 ml) was then added. After stirring for another 1 h, the mixture was cooling slowly to room temperature, and kept stirring overnight. To the mixture was added dichloromethane (20 ml) and stirred for 1 h. The solvent was evaporated under reduced pressure. The crude products were purified by columnchromatography (silica gel) using n-hexane/dichloromethane as eluent. The title compound was obtained as white solid in 31% yield. Colorless single crystals were grown from a CH_2_Cl_2_ solution of the compound.

### Refinement

H atoms were positioned geometrically and treated as riding, with C—H = 0.93 (aromatic), 0.97 (methylene) and 0.98 Å (methine), and refined in riding mode with U_iso_(H) = 1.2U_eq_(C).

### Crystal data

**Table:**

C_28_H_21_Cl	*F*_000_ = 824
*M**_r_* = 392.90	*D*_x_ = 1.290 Mg m^−^^3^
Monoclinic, *P*2_1_/*c*	Mo *K*α radiation λ = 0.71073 Å
Hall symbol: -P 2ybc	Cell parameters from 12994 reflections
*a* = 8.4346 (17) Å	θ = 3.1–27.4º
*b* = 26.368 (5) Å	µ = 0.20 mm^−^^1^
*c* = 9.1162 (18) Å	*T* = 298 (2) K
β = 94.08 (3)º	Chunk, colorless
*V* = 2022.3 (7) Å^3^	0.35 × 0.29 × 0.22 mm
*Z* = 4	

### Data collection

**Table:**

Bruker SMART 1000 CCD area-detector diffractometer	2747 reflections with *I* > 2σ(*I*)
Radiation source: fine-focus sealed tube	*R*_int_ = 0.025
Monochromator: graphite	θ_max_ = 25.3º
*T* = 298(2) K	θ_min_ = 3.1º
φ and ω scans	*h* = −10→10
Absorption correction: none	*k* = −31→31
16094 measured reflections	*l* = −10→10
3646 independent reflections	

### Refinement

**Table:**

Refinement on *F*^2^	Hydrogen site location: inferred from neighbouring sites
Least-squares matrix: full	H-atom parameters constrained
*R*[*F*^2^ > 2σ(*F*^2^)] = 0.040	*w* = 1/[σ^2^(*F*_o_^2^) + (0.0595*P*)^2^ + 0.3188*P*] where *P* = (*F*_o_^2^ + 2*F*_c_^2^)/3
*wR*(*F*^2^) = 0.133	(Δ/σ)_max_ < 0.001
*S* = 1.08	Δρ_max_ = 0.22 e Å^−^^3^
3646 reflections	Δρ_min_ = −0.25 e Å^−^^3^
263 parameters	Extinction correction: SHELXTL (Sheldrick, 2008), Fc^*^=kFc[1+0.001xFc^2^λ^3^/sin(2θ)]^-1/4^
Primary atom site location: structure-invariant direct methods	Extinction coefficient: 0.011 (2)
Secondary atom site location: difference Fourier map	

### Special details

**Table:**

Experimental. ^1^HNMR (500 MHz, δ in p.p.m., CDCl_3_): 2.90 (d, 2H, *J* = 5.5 Hz), 3.19 (t, 1H,*J*= 4.5 Hz), 3.86 (s, 2H), 6.62 (d, 2H, *J* = 7.0 Hz), 7.02 (t, 2H, *J*= 7.5 Hz), 7.19 (t, 2H, *J* = 7.5 Hz), 7.39 (t, 2H, *J* = 7.5 Hz), 7.46 (t, 2H, *J* = 7.5 Hz), 7.54 (d, 2H,*J* = 7.5 Hz), 7.67 (d, 2H, *J* = 7.5 Hz), 7.78 (d, 2H, *J*= 7.0 Hz); ^13^C NMR (125 MHz, δ in p.p.m., CDCl_3_): 40.64, 44.45, 53.35, 55.55, 119.52, 120.75, 125.06, 125.18, 126.86, 126.92, 127.66,128.74, 140.70, 141.48, 146.99, 148.22; MS (EI): calcd for C_28_H_21_Cl, 392; found: 392 (*M*^+^), 356, 191 (100), 179, 165, 152.
Geometry. All e.s.d.'s (except the e.s.d. in the dihedral angle between two l.s. planes) are estimated using the full covariance matrix. The cell e.s.d.'s are taken into account individually in the estimation of e.s.d.'s in distances, angles and torsion angles; correlations between e.s.d.'s in cell parameters are only used when they are defined by crystal symmetry. An approximate (isotropic) treatment of cell e.s.d.'s is used for estimating e.s.d.'s involving l.s. planes.
Refinement. Refinement of *F*^2^ against ALL reflections. The weighted *R*-factor *wR* and goodness of fit *S* are based on *F*^2^, conventional *R*-factors *R* are based on *F*, with *F* set to zero for negative *F*^2^. The threshold expression of *F*^2^ > σ(*F*^2^) is used only for calculating *R*-factors(gt) *etc*. and is not relevant to the choice of reflections for refinement. *R*-factors based on *F*^2^ are statistically about twice as large as those based on *F*, and *R*- factors based on ALL data will be even larger.

### Fractional atomic coordinates and isotropic or equivalent isotropic displacement parameters (Å^2^)

**Table:**

	*x*	*y*	*z*	*U*_iso_*/*U*_eq_	
Cl1	0.14417 (6)	0.73515 (2)	0.46199 (7)	0.0815 (2)	
C1	0.38752 (16)	0.68247 (6)	0.60052 (19)	0.0421 (4)	
C2	0.30669 (16)	0.65243 (5)	0.72229 (18)	0.0407 (4)	
C3	0.16272 (18)	0.63563 (7)	0.7173 (2)	0.0546 (5)	
H3	0.0926	0.6402	0.6349	0.066*	
C4	0.1160 (2)	0.60914 (7)	0.8475 (3)	0.0671 (6)	
H4	0.0121	0.5971	0.8434	0.080*	
C5	0.2094 (2)	0.59950 (7)	0.9791 (3)	0.0651 (5)	
H5	0.1692	0.5821	1.0571	0.078*	
C6	0.3524 (2)	0.61602 (7)	0.9853 (2)	0.0547 (5)	
H6	0.4216	0.6113	1.0682	0.066*	
C7	0.40065 (17)	0.64228 (5)	0.85735 (18)	0.0410 (4)	
C8	0.54497 (17)	0.66472 (6)	0.83543 (19)	0.0427 (4)	
C9	0.6747 (2)	0.66400 (7)	0.9329 (2)	0.0581 (5)	
H9	0.6787	0.6472	1.0228	0.070*	
C10	0.7950 (2)	0.69009 (8)	0.8846 (3)	0.0731 (7)	
H10	0.8899	0.6913	0.9430	0.088*	
C11	0.7859 (2)	0.71698 (9)	0.7450 (3)	0.0778 (7)	
H11	0.8749	0.7353	0.7215	0.093*	
C12	0.6578 (2)	0.71744 (7)	0.6465 (3)	0.0635 (5)	
H12	0.6545	0.7349	0.5576	0.076*	
C13	0.53712 (17)	0.69020 (6)	0.6907 (2)	0.0455 (4)	
C14	0.47723 (18)	0.60085 (6)	0.46017 (19)	0.0461 (4)	
H14	0.5053	0.5943	0.5646	0.055*	
C15	0.38570 (18)	0.55633 (6)	0.39464 (19)	0.0460 (4)	
C16	0.2451 (2)	0.54170 (7)	0.4245 (2)	0.0573 (5)	
H16	0.1867	0.5592	0.4909	0.069*	
C17	0.1854 (2)	0.49837 (7)	0.3523 (2)	0.0637 (5)	
H17	0.0848	0.4874	0.3737	0.076*	
C18	0.2642 (2)	0.47010 (7)	0.2507 (3)	0.0659 (5)	
H18	0.2167	0.4416	0.2063	0.079*	
C19	0.4057 (2)	0.48432 (7)	0.2191 (2)	0.0593 (5)	
H19	0.4628	0.4668	0.1516	0.071*	
C20	0.46626 (18)	0.52716 (6)	0.29204 (19)	0.0473 (4)	
C21	0.61091 (18)	0.55048 (7)	0.28235 (19)	0.0497 (4)	
C22	0.7292 (2)	0.53544 (8)	0.1985 (2)	0.0626 (5)	
H22	0.7211	0.5070	0.1383	0.075*	
C23	0.8541 (2)	0.56413 (10)	0.2096 (3)	0.0737 (6)	
H23	0.9398	0.5561	0.1552	0.088*	
C24	0.8643 (2)	0.60722 (9)	0.3016 (3)	0.0766 (6)	
H24	0.9573	0.6262	0.3044	0.092*	
C25	0.7467 (2)	0.62294 (9)	0.3868 (2)	0.0666 (6)	
H25	0.7561	0.6515	0.4466	0.080*	
C26	0.61912 (18)	0.59411 (7)	0.37704 (19)	0.0501 (4)	
C27	0.40233 (18)	0.65354 (6)	0.44306 (19)	0.0472 (4)	
H27A	0.2972	0.6503	0.3934	0.057*	
H27B	0.4661	0.6741	0.3816	0.057*	
C28	0.3207 (2)	0.73528 (7)	0.5688 (2)	0.0576 (5)	
H28A	0.3983	0.7549	0.5193	0.069*	
H28B	0.3045	0.7520	0.6613	0.069*	

### Atomic displacement parameters (Å^2^)

**Table:**

	*U*^11^	*U*^22^	*U*^33^	*U*^12^	*U*^13^	*U*^23^
Cl1	0.0610 (3)	0.0870 (4)	0.0956 (5)	0.0332 (3)	−0.0002 (3)	0.0213 (3)
C1	0.0348 (7)	0.0412 (8)	0.0506 (10)	0.0036 (6)	0.0054 (7)	0.0058 (7)
C2	0.0311 (7)	0.0375 (8)	0.0540 (10)	0.0019 (6)	0.0063 (7)	−0.0014 (7)
C3	0.0342 (8)	0.0576 (10)	0.0720 (13)	−0.0010 (7)	0.0043 (8)	−0.0049 (9)
C4	0.0415 (9)	0.0611 (11)	0.1018 (17)	−0.0098 (8)	0.0271 (10)	−0.0070 (11)
C5	0.0605 (11)	0.0605 (11)	0.0774 (15)	−0.0026 (9)	0.0271 (10)	0.0139 (10)
C6	0.0519 (10)	0.0536 (10)	0.0593 (12)	0.0054 (8)	0.0092 (8)	0.0090 (9)
C7	0.0390 (8)	0.0344 (7)	0.0503 (10)	0.0048 (6)	0.0075 (7)	0.0000 (7)
C8	0.0333 (7)	0.0382 (8)	0.0563 (11)	0.0045 (6)	0.0020 (7)	−0.0065 (7)
C9	0.0454 (9)	0.0554 (10)	0.0720 (13)	0.0096 (8)	−0.0072 (9)	−0.0139 (9)
C10	0.0352 (9)	0.0727 (13)	0.1100 (19)	0.0001 (9)	−0.0045 (10)	−0.0327 (13)
C11	0.0392 (9)	0.0748 (13)	0.121 (2)	−0.0183 (9)	0.0167 (11)	−0.0319 (14)
C12	0.0499 (10)	0.0556 (10)	0.0871 (15)	−0.0133 (8)	0.0188 (10)	−0.0032 (10)
C13	0.0351 (7)	0.0404 (8)	0.0616 (11)	−0.0011 (6)	0.0087 (7)	−0.0031 (8)
C14	0.0401 (8)	0.0526 (9)	0.0453 (10)	0.0095 (7)	0.0015 (7)	0.0017 (7)
C15	0.0412 (8)	0.0476 (9)	0.0488 (10)	0.0104 (7)	0.0018 (7)	0.0071 (7)
C16	0.0467 (9)	0.0584 (10)	0.0678 (13)	0.0059 (8)	0.0118 (8)	0.0026 (9)
C17	0.0487 (10)	0.0574 (11)	0.0854 (15)	−0.0004 (8)	0.0075 (10)	0.0087 (11)
C18	0.0600 (11)	0.0501 (10)	0.0869 (15)	0.0005 (9)	0.0005 (10)	−0.0018 (10)
C19	0.0562 (10)	0.0511 (10)	0.0705 (13)	0.0136 (8)	0.0047 (9)	−0.0029 (9)
C20	0.0426 (8)	0.0471 (9)	0.0519 (11)	0.0125 (7)	0.0002 (7)	0.0067 (8)
C21	0.0418 (8)	0.0574 (10)	0.0496 (10)	0.0171 (7)	0.0013 (7)	0.0059 (8)
C22	0.0470 (10)	0.0744 (12)	0.0663 (13)	0.0196 (9)	0.0045 (9)	−0.0018 (10)
C23	0.0406 (10)	0.1029 (17)	0.0786 (15)	0.0206 (10)	0.0110 (9)	0.0027 (13)
C24	0.0348 (9)	0.1053 (17)	0.0897 (17)	0.0044 (10)	0.0036 (10)	−0.0003 (14)
C25	0.0412 (9)	0.0843 (14)	0.0737 (14)	0.0025 (9)	−0.0006 (9)	−0.0097 (11)
C26	0.0349 (8)	0.0629 (10)	0.0515 (11)	0.0120 (7)	−0.0029 (7)	0.0022 (8)
C27	0.0383 (8)	0.0532 (9)	0.0499 (10)	0.0097 (7)	0.0024 (7)	0.0078 (8)
C28	0.0527 (10)	0.0496 (10)	0.0714 (13)	0.0093 (8)	0.0104 (9)	0.0101 (9)

### Geometric parameters (Å, °)

**Table:**

Cl1—C28	1.720 (2)	C14—C27	1.530 (2)
C1—C13	1.470 (2)	C14—H14	0.9800
C1—C28	1.522 (2)	C15—C16	1.294 (2)
C1—C2	1.560 (2)	C15—C20	1.421 (2)
C1—C27	1.638 (2)	C16—C17	1.395 (3)
C2—C3	1.290 (2)	C16—H16	0.9300
C2—C7	1.441 (2)	C17—C18	1.394 (3)
C3—C4	1.455 (3)	C17—H17	0.9300
C3—H3	0.9300	C18—C19	1.302 (3)
C4—C5	1.411 (3)	C18—H18	0.9300
C4—H4	0.9300	C19—C20	1.390 (3)
C5—C6	1.280 (3)	C19—H19	0.9300
C5—H5	0.9300	C20—C21	1.375 (2)
C6—C7	1.440 (2)	C21—C22	1.358 (2)
C6—H6	0.9300	C21—C26	1.437 (3)
C7—C8	1.381 (2)	C22—C23	1.295 (3)
C8—C9	1.360 (2)	C22—H22	0.9300
C8—C13	1.478 (2)	C23—C24	1.411 (3)
C9—C10	1.326 (3)	C23—H23	0.9300
C9—H9	0.9300	C24—C25	1.367 (3)
C10—C11	1.454 (4)	C24—H24	0.9300
C10—H10	0.9300	C25—C26	1.315 (3)
C11—C12	1.355 (3)	C25—H25	0.9300
C11—H11	0.9300	C27—H27A	0.9700
C12—C13	1.331 (2)	C27—H27B	0.9700
C12—H12	0.9300	C28—H28A	0.9700
C14—C26	1.472 (2)	C28—H28B	0.9700
C14—C15	1.505 (2)		
			
C13—C1—C28	105.72 (13)	C16—C15—C20	117.73 (17)
C13—C1—C2	94.22 (12)	C16—C15—C14	126.93 (17)
C28—C1—C2	115.17 (13)	C20—C15—C14	115.32 (14)
C13—C1—C27	116.01 (12)	C15—C16—C17	116.78 (18)
C28—C1—C27	108.04 (14)	C15—C16—H16	121.6
C2—C1—C27	116.85 (12)	C17—C16—H16	121.6
C3—C2—C7	115.17 (16)	C18—C17—C16	125.34 (18)
C3—C2—C1	127.39 (16)	C18—C17—H17	117.3
C7—C2—C1	117.44 (13)	C16—C17—H17	117.3
C2—C3—C4	116.40 (17)	C19—C18—C17	118.73 (19)
C2—C3—H3	121.8	C19—C18—H18	120.6
C4—C3—H3	121.8	C17—C18—H18	120.6
C5—C4—C3	127.81 (16)	C18—C19—C20	116.08 (18)
C5—C4—H4	116.1	C18—C19—H19	122.0
C3—C4—H4	116.1	C20—C19—H19	122.0
C6—C5—C4	116.31 (19)	C21—C20—C19	129.09 (16)
C6—C5—H5	121.8	C21—C20—C15	105.57 (15)
C4—C5—H5	121.8	C19—C20—C15	125.33 (16)
C5—C6—C7	116.61 (19)	C22—C21—C20	126.61 (18)
C5—C6—H6	121.7	C22—C21—C26	124.45 (17)
C7—C6—H6	121.7	C20—C21—C26	108.94 (15)
C8—C7—C6	128.77 (16)	C23—C22—C21	114.3 (2)
C8—C7—C2	103.51 (14)	C23—C22—H22	122.8
C6—C7—C2	127.70 (15)	C21—C22—H22	122.8
C9—C8—C7	125.19 (17)	C22—C23—C24	122.10 (19)
C9—C8—C13	124.93 (16)	C22—C23—H23	118.9
C7—C8—C13	109.88 (14)	C24—C23—H23	118.9
C10—C9—C8	112.0 (2)	C25—C24—C23	124.5 (2)
C10—C9—H9	124.0	C25—C24—H24	117.8
C8—C9—H9	124.0	C23—C24—H24	117.8
C9—C10—C11	123.34 (18)	C26—C25—C24	114.1 (2)
C9—C10—H10	118.3	C26—C25—H25	122.9
C11—C10—H10	118.3	C24—C25—H25	122.9
C12—C11—C10	125.04 (19)	C25—C26—C21	120.52 (17)
C12—C11—H11	117.5	C25—C26—C14	125.89 (18)
C10—C11—H11	117.5	C21—C26—C14	113.56 (15)
C13—C12—C11	112.7 (2)	C14—C27—C1	112.98 (13)
C13—C12—H12	123.7	C14—C27—H27A	109.0
C11—C12—H12	123.7	C1—C27—H27A	109.0
C12—C13—C1	123.32 (17)	C14—C27—H27B	109.0
C12—C13—C8	121.94 (17)	C1—C27—H27B	109.0
C1—C13—C8	114.73 (13)	H27A—C27—H27B	107.8
C26—C14—C15	96.56 (14)	C1—C28—Cl1	113.56 (13)
C26—C14—C27	113.72 (15)	C1—C28—H28A	108.9
C15—C14—C27	118.13 (13)	Cl1—C28—H28A	108.9
C26—C14—H14	109.2	C1—C28—H28B	108.9
C15—C14—H14	109.2	Cl1—C28—H28B	108.9
C27—C14—H14	109.2	H28A—C28—H28B	107.7
			
C13—C1—C2—C3	176.17 (17)	C26—C14—C15—C20	1.81 (17)
C28—C1—C2—C3	66.6 (2)	C27—C14—C15—C20	123.19 (16)
C27—C1—C2—C3	−61.8 (2)	C20—C15—C16—C17	−0.1 (3)
C13—C1—C2—C7	−3.14 (16)	C14—C15—C16—C17	−178.46 (16)
C28—C1—C2—C7	−112.70 (16)	C15—C16—C17—C18	−0.5 (3)
C27—C1—C2—C7	118.88 (14)	C16—C17—C18—C19	0.4 (3)
C7—C2—C3—C4	0.1 (2)	C17—C18—C19—C20	0.4 (3)
C1—C2—C3—C4	−179.23 (15)	C18—C19—C20—C21	179.31 (19)
C2—C3—C4—C5	−0.1 (3)	C18—C19—C20—C15	−1.0 (3)
C3—C4—C5—C6	0.1 (3)	C16—C15—C20—C21	−179.38 (16)
C4—C5—C6—C7	−0.1 (3)	C14—C15—C20—C21	−0.83 (19)
C5—C6—C7—C8	178.42 (17)	C16—C15—C20—C19	0.9 (3)
C5—C6—C7—C2	0.2 (3)	C14—C15—C20—C19	179.43 (16)
C3—C2—C7—C8	−178.76 (14)	C19—C20—C21—C22	−1.2 (3)
C1—C2—C7—C8	0.64 (17)	C15—C20—C21—C22	179.06 (17)
C3—C2—C7—C6	−0.2 (2)	C19—C20—C21—C26	179.09 (17)
C1—C2—C7—C6	179.23 (15)	C15—C20—C21—C26	−0.64 (18)
C6—C7—C8—C9	3.1 (3)	C20—C21—C22—C23	−179.87 (19)
C2—C7—C8—C9	−178.30 (15)	C26—C21—C22—C23	−0.2 (3)
C6—C7—C8—C13	−176.25 (15)	C21—C22—C23—C24	0.0 (3)
C2—C7—C8—C13	2.33 (16)	C22—C23—C24—C25	0.1 (4)
C7—C8—C9—C10	−177.88 (16)	C23—C24—C25—C26	0.1 (3)
C13—C8—C9—C10	1.4 (2)	C24—C25—C26—C21	−0.3 (3)
C8—C9—C10—C11	1.2 (3)	C24—C25—C26—C14	177.61 (17)
C9—C10—C11—C12	−2.2 (3)	C22—C21—C26—C25	0.4 (3)
C10—C11—C12—C13	0.1 (3)	C20—C21—C26—C25	−179.91 (18)
C11—C12—C13—C1	−176.77 (16)	C22—C21—C26—C14	−177.76 (16)
C11—C12—C13—C8	2.5 (3)	C20—C21—C26—C14	2.0 (2)
C28—C1—C13—C12	−58.6 (2)	C15—C14—C26—C25	179.82 (19)
C2—C1—C13—C12	−176.21 (16)	C27—C14—C26—C25	55.1 (3)
C27—C1—C13—C12	61.1 (2)	C15—C14—C26—C21	−2.17 (17)
C28—C1—C13—C8	122.11 (15)	C27—C14—C26—C21	−126.84 (16)
C2—C1—C13—C8	4.48 (15)	C26—C14—C27—C1	−123.54 (15)
C27—C1—C13—C8	−118.20 (15)	C15—C14—C27—C1	124.34 (16)
C9—C8—C13—C12	−3.6 (3)	C13—C1—C27—C14	58.35 (18)
C7—C8—C13—C12	175.79 (16)	C28—C1—C27—C14	176.78 (13)
C9—C8—C13—C1	175.74 (15)	C2—C1—C27—C14	−51.45 (17)
C7—C8—C13—C1	−4.88 (18)	C13—C1—C28—Cl1	179.50 (12)
C26—C14—C15—C16	−179.80 (18)	C2—C1—C28—Cl1	−77.98 (18)
C27—C14—C15—C16	−58.4 (2)	C27—C1—C28—Cl1	54.69 (17)

### Hydrogen-bond geometry (Å, °)

**Table:**

*D*—H···*A*	*D*—H	H···*A*	*D*···*A*	*D*—H···*A*
C27—H27A···Cl1	0.97	2.68	3.075 (2)	105
C10—H10···Cl1^i^	0.93	2.89	3.573 (2)	131

Symmetry codes: (i) *x*+1, −*y*+3/2, *z*+1/2.
